# Cancer Cell Membrane-Coated NPs as a Biomimetic Strategy for Precision Tumor Therapy

**DOI:** 10.3390/pharmaceutics17101322

**Published:** 2025-10-11

**Authors:** Junyi Lin, Wei Li, Alaa R. Aboushanab, Jingjing Sun

**Affiliations:** 1 Department of Pharmaceutical Sciences, College of Pharmacy, University of Nebraska Medical Center, Omaha, NE 68106, USA; 2 Fred & Pamela Buffett Cancer Center, University of Nebraska Medical Center, Omaha, NE 68106, USA

**Keywords:** biomimetic nanocarrier, cancer cell membrane, NPs, drug delivery, cancer vaccines

## Abstract

Cancer treatment remains challenging due to the complexity of the tumor microenvironment, which promotes tumor heterogeneity and contributes to the development of multidrug resistance, ultimately hindering drug delivery and reducing therapeutic efficacy. In recent years, biomimetic nanocarriers have emerged as promising tools to address these challenges. Among them, cancer cell membrane (CCM)-coated nanoparticles (CCM-NPs) have attracted increasing attention due to their unique advantages, including homologous targeting, prolonged circulation mediated by self-recognition, and enhanced tumor penetration. Moreover, CCM-NPs can serve as versatile platforms for tumor vaccines by leveraging their inherent tumor-associated antigens and immunomodulatory potential. By leveraging CCMs to functionalize NPs, researchers have developed innovative approaches to improve drug delivery, enhance tumor immunotherapy, and optimize cancer vaccine efficacy. Despite these advancements, a comprehensive review summarizing the latest progress in CCM-based biomimetic nanocarriers for tumor treatment is lacking. This review integrates recent advances in CCM-NPs for targeted drug delivery and cancer vaccination, and discusses their fabrication, characterization, mechanisms and applications across multiple cancer types, which provides timely insights to guide their future development in precision tumor therapy.

## 1. Introduction

Cancer remains one of the most critical public health and economic challenges of the 21st century, accounting for nearly one in six deaths (16.8%) globally and 22.8% of all noncommunicable disease (NCD)-related deaths. It is responsible for 30.3% of premature deaths from NCDs among individuals aged 30–70 years and ranks among the top three causes of death in this age group across 177 of 183 countries [1]. Beyond its impact on life expectancy, cancer imposes significant societal and economic burdens, with variations depending on cancer type, geographic region, and gender [2]. A recent study highlighted the disproportionate impact of cancer mortality among women, estimating that in 2020 alone, one million children lost their mothers to cancer, with nearly half of these maternal deaths caused by breast or cervical cancer [3]. Chemotherapy remains a primary approach for treating cancer, particularly for inoperable or metastatic tumors. However, survival rates often remain stagnant even after treatment due to limitations such as systemic toxicity, drug resistance, and poor tumor specificity. Immunotherapy has revolutionized cancer treatment by harnessing the body’s immune system to recognize and eliminate tumor cells. Therapeutic approaches such as immune checkpoint inhibitors (ICIs), chimeric antigen receptor (CAR)-T cell therapy, and cancer vaccines have significantly improved outcomes in certain malignancies, particularly hematologic cancers and tumors with high immunogenicity [4,5]. Despite its success, poor T-cell infiltration, antigen heterogeneity, and T-cell exhaustion limit treatment efficacy in some tumor types, while severe immune-related toxicities such as cytokine release syndrome (CRS) and autoimmune reactions pose safety concerns [6,7].

Nanotechnology is a powerful tool for overcoming the limitations of conventional cancer treatments by enabling controlled drug release, enhanced bioavailability, and improved tumor accumulation through various nanoparticle (NP) platforms. Among various NP-based approaches, surface-modified NPs, such as polyethylene glycol (PEG)-coated NPs and ligand-functionalized nanocarriers, have been widely explored to improve circulation time and tumor accumulation. Although PEGylation helps NPs evade immune recognition, it suffers from limitations such as anti-PEG immune responses, lack of active tumor targeting, and eventual clearance from the body [8]. Similarly, ligand-targeted NPs, designed for cell-specific internalization, face challenges due to tumor antigen heterogeneity, opsonization by serum proteins, and unintended immune uptake, which reduce their targeting efficiency [9]. These drawbacks necessitate the development of next-generation biomimetic nanocarriers with improved specificity and therapeutic potential.

In recent years, cell membrane-coated NPs have developed as a promising strategy to address these challenges [10]. By camouflaging NPs with natural cell membranes, this biomimetic approach enhances immune evasion, tumor targeting, and biocompatibility [11]. Different membrane sources have been explored, each with characteristic strengths and drawbacks (Table 1). Red blood cell (RBC) membranes provide excellent immune evasion and prolonged circulation but lack tumor specificity [12]. Platelet membranes display affinity toward damaged vasculature and circulating tumor cells, which may help limit metastasis, though pro-thrombotic activity remains a concern [13]. Immune cell membranes, such as those derived from macrophages, T cells, or NK cells, can direct NPs to inflammatory or tumor microenvironments and contribute to immune modulation, but functional variability and possible immunogenicity limit their use [14]. Stem cell membranes show natural tropism toward tumors and injured tissues and confer immune evasion, yet issues of safety and large-scale preparation remain challenging [15,16].

Among these strategies, cancer cell membrane-coated NPs (CCM-NPs) are particularly attractive for oncology applications. Unlike other membrane types, CCM-NPs retain tumor-specific adhesion molecules and homotypic recognition properties, allowing selective interaction with tumor tissues. In addition, they preserve immune evasion proteins such as CD47, which enhance circulation, biodistribution, and tumor accumulation [17]. These features make CCM-NPs especially suited for precision cancer therapy. Furthermore, the ability of CCM-NPs to retain tumor-associated antigens also makes them attractive candidates for cancer vaccine development, as they can co-deliver immunostimulatory agents (e.g., adjuvants or checkpoint blockers) to effectively prime anti-tumor immunity [18]. Despite these advancements, several challenges remain, including scalability, standardization of membrane isolation, and regulatory hurdles for clinical translation [19].

Unlike earlier reviews that broadly summarize diverse cell membrane-coated nanoparticles [11] or focus on imaging and therapy in specific cancers [20], this review provides a comprehensive overview of recent advances in CCM-NPs, focusing on emerging applications as cancer vaccines and precision drug delivery systems. Their mechanisms of homotypic targeting, immune evasion, and tumor microenvironment penetration will be discussed. In addition, different preparation strategies and characterization methods will be summarized, and recent applications of CCM-NPs across multiple cancer types will be highlighted. Taken together, this review offers timely insights that may inform the rational design of CCM-NPs for precision tumor therapy.

## 2. Biological Functions and Mechanisms of CCM-Coated NPs

### 2.1. Enhancing Drug Delivery by Targeting Tumor, Avoiding Phagocytosis and Penetrating Tumor Microenvironment (TME)

CCM-NPs possess distinctive biological properties that significantly enhance their therapeutic potential in oncology. A key advantage of CCM-NPs lies in their ability to selectively target tumors via homotypic interactions. Cancer cells communicate and aggregate via adhesion molecules such as E-cadherin, *N*-cadherin, galectin-3, integrins, and selectins. When NPs are coated with cancer cell membranes, they display the same molecules, which allows them to bind selectively to tumor cells and to penetrate clusters of tumor tissue through self-recognition. Scully et al. engineered PLGA nanoparticles coated with membranes from 4T1 murine mammary cancer cells to achieve selective targeting of triple-negative breast cancer (TNBC) [21]. These nanoparticles were loaded with ABT-737, a Bcl-2 inhibitor, exploiting Bcl-2 blockade as a potential therapeutic strategy against this highly aggressive cancer. The incorporation of 4T1-derived cancer cell membranes greatly improved the tumor-homing ability of the nanoparticles, resulting in enhanced accumulation at tumor sites and more effective delivery of ABT-737 [21]. Similarly, Harris et al. developed a targeted chemotherapeutic strategy for acute myeloid leukemia (AML) by coating polymeric nanoparticles carrying doxorubicin (DOX) with membranes obtained from human AML cells (CHRF-288-11 line). Their results showed that these cell membrane-coated nanoparticles could induce apoptosis in up to 80% of target cells, representing a significant improvement over conventional DOX administration [22].

Another critical feature of CCM-NPs is their ability to evade immune clearance, a challenge that conventional NPs often struggle with. The immune system rapidly recognizes and eliminates synthetic NPs, especially those without specialized coatings. In contrast, CCM-NPs retain the CD47 protein, which interacts with signal-regulatory protein alpha (SIRPα) on macrophages, sending a “don’t eat me” signal that helps them avoid phagocytosis [23]. As a result, CCM-NPs exhibit longer circulation times in the bloodstream compared to PEGylated NPs, which can still be recognized and cleared by the immune system due to the development of anti-PEG antibodies [24,25]. This prolonged circulation not only increases the probability of tumor accumulation but also improves the area under the concentration–time curve (AUC), resulting in higher systemic drug exposure. In addition, CCM-NPs demonstrate preferential tumor uptake through homotypic recognition of adhesion molecules while showing reduced accumulation in clearance organs such as the liver and spleen compared with uncoated nanoparticles. These combined effects on pharmacokinetics and tissue distribution contribute to enhanced therapeutic efficacy and a more favorable safety profile.

Beyond their targeting and immune evasion properties, CCM-NPs are also highly effective at penetrating the TME, which is often a major barrier to drug delivery. Solid tumors develop dense extracellular matrices (ECM), abnormal vasculature, and high interstitial fluid pressure, all of which make it difficult for therapeutics to reach deep into tumor tissue. Tumor cells naturally remodel and migrate through the TME by engaging ECM proteins such as fibronectin, collagen, and laminin. CCM-NPs inherit part of this machinery, which can help them better interact with the dense tumor stroma, allowing for better penetration compared to conventional NPs. For example, Fang et al. showed enhanced infiltration into 3D tumor spheroids by cancer cell membrane-coated DOX nanoparticles compared to uncoated controls [26]. Similarly, Nie et al. reported that yolk-shell CCM-NPs significantly improved tumor penetration and intracellular trafficking, augmenting chemotherapy efficacy [27]. Furthermore, Chen et al. developed CCM-coated PLGA nanospheres that actively accumulated in tumors under PA/MR imaging guidance, facilitating deeper TME penetration and enhancing therapeutic outcomes [28]. Some studies suggest that modifying CCM-NPs with ECM-degrading enzymes, such as matrix metalloproteinases (MMPs), can further enhance their ability to break through these barriers, leading to higher drug accumulation in the tumor core [29,30].

### 2.2. Serving as Biomimetic Nanovaccines and Cancer Immunotherapy Platforms

CCMs are abundant in tumor-associated antigens and neoantigens, making them valuable components for designing cancer vaccines and other therapeutic approaches [10]. CCM-NPs provide an ideal platform for nanovaccines, as the combination of the membrane coating and an immunostimulatory nanoparticle core can elicit a strong antitumor immune response. This dual capability of tumor targeting and immune activation was first demonstrated in 2014 [26]. Dendritic cells (DCs) can efficiently recognize these NPs, process the antigens, and present them to T cells, triggering a robust anti-tumor immune response [31,32]. This makes CCM-NPs highly promising for use in combination with ICIs, such as PD-1/PD-L1 blockade therapy, to reactivate exhausted T cells in the TME. In addition, CCM-NPs are being investigated to enhance the infiltration and persistence of CAR-T cells in solid tumors, overcoming one of the major challenges of current CAR-T treatments.

To enhance vaccine efficacy, CCM-NPs are frequently engineered with immune adjuvants (Table 2). For example, Johnson et al. developed an acute myeloid leukemia (AML) nanovaccine using AML cell membranes to coat PLGA nanoparticles encapsulating the immunostimulatory adjuvant CpG oligodeoxynucleotide 1826 [33]. This construct co-delivered multiple TAAs and CpG to antigen-presenting cells (APCs), promoting DC activation, co-stimulatory signaling, and leukemia-specific immune responses, thereby boosting anti-leukemia efficacy. Similarly, Zhao et al. fused 4T1 breast cancer cell membranes with bone marrow-derived DC membranes to form hybrid CCMs, encapsulating R837-loaded mesoporous silica nanoparticles (R837@HM-NPs) [34]. The NPs effectively stimulated DC maturation, enhanced antigen presentation, and induced DCs to secrete large amounts of pro-inflammatory cytokines. In vivo experiments indicated that R837@HM-NPs combined with anti-PD-1 therapy could modulate the tumor-immunosuppressive microenvironment, effectively inhibiting tumor growth and recurrence, and prolonging the survival of tumor-bearing mice.

Other studies have further modified or engineered CCMs to overcome immune evasion and improve APC targeting. Wang et al. constructed a colorectal cancer CCM-coated layered double hydroxide (LDH) nanovaccine (LGCMB), modified with bovine serum albumin (BSA) and mannose to block immune-evasive proteins and enhance DC uptake [35]. This system promoted CD8^+^ T-cell infiltration and suppressed tumor growth in murine models. Li et al. enriched tumor membranes with neoantigens via IFN-γ stimulation and coated them onto a STING-activating polymer (PC7A), generating the AECM@PC7A vaccine [36]. This AECM@PC7A nanovaccine effectively stimulates DC maturation and cross-dressing-based antigen presentation, leading to robust CD8^+^ T-cell activation and poly-epitopic antitumor immune responses. The strategy bypasses the need for precise neoantigen identification and demonstrates potent efficacy across multiple models, including post-surgical recurrence models and humanized xenograft models.

**Figure 1:**
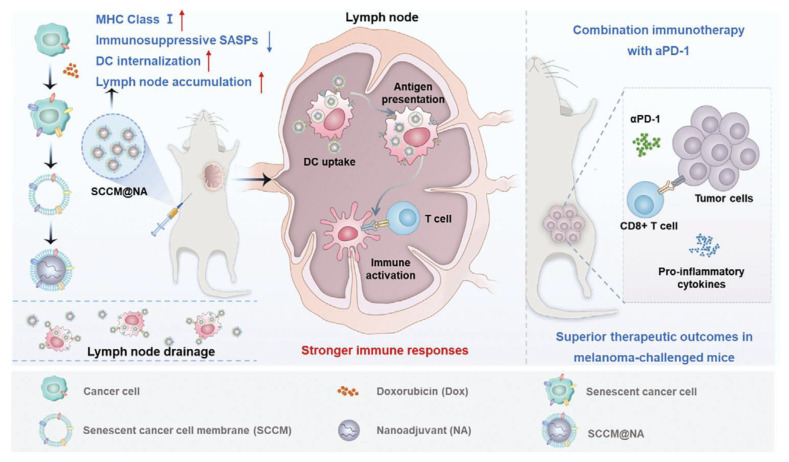
A biomimetic nanovaccine constructed from senescent tumor cell membranes and nanoscale adjuvants enhanced dendritic cell uptake, lymph node targeting, and immune activation compared with traditional or ICD-derived vaccines. Combined with anti–PD-1 therapy, it elicited potent antitumor immunity and improved therapeutic efficacy in a melanoma model while reducing side effects. Adapted with permission. Ref. [37] Copyright 2024, Adv Sci (Weinh).

Senescent tumor cells possess high immunogenicity and hold great potential in cancer immunotherapy. However, the efficacy of senescent tumor cell-based vaccines is still limited by the immunosuppressive senescence-associated secretory phenotype (SASP) and the inherent tumorigenic risk. To overcome these challenges, Shao et al. [37] collaboratively reported a biomimetic nanovaccine based on senescent tumor cell membranes to enhance cancer immunotherapy. By integrating senescent tumor cell membranes with nanoscale adjuvants, the team constructed a biomimetic nanovaccine that outperformed traditional senescent tumor cell vaccines in promoting DC uptake, improving lymph node targeting, and activating immune responses. Compared with biomimetic nanovaccines derived from immunogenic cell death (ICD)-induced tumor cells, the senescent cell membrane-based nanovaccine more effectively promoted DC maturation. When combined with anti-PD-1 therapy, it elicited a potent antitumor immune response in a melanoma mouse model, enhancing therapeutic efficacy while reducing side effects. This study proposes a novel design strategy for biomimetic nanovaccines that improves vaccine immunogenicity and addresses the potential risks associated with senescent tumor cell vaccines (Figure 1).

Tumor vaccines developed from autologous tumor cell membranes hold great promise for more effective immunotherapy tailored to individual patients. Nevertheless, in clinical practice, patients eligible for autologous tumor membrane vaccines have often undergone multiple treatments, including chemotherapy, which may alter antigen presentation or reduce antigen immunogenicity on tumor membranes, ultimately impacting vaccine efficacy. Recent work has shown that neoadjuvant chemotherapy (e.g., doxorubicin, liposomal doxorubicin) can enhance the immunogenicity of tumor membranes by upregulating immune-related proteins, thereby improving the efficacy of subsequent autologous CCM-based vaccines [38]. Nanovaccines derived from liposomal doxorubicin-treated tumor membranes exhibited stronger antigen presentation and stronger antitumor immune responses, effectively suppressing recurrence and metastasis while prolonging survival in various murine models. This study not only provides valuable insights for the clinical translation of autologous tumor membrane vaccines but also highlights the significance and potential advantages of integrating CCMs with neoadjuvant chemotherapy for cancer immunotherapy.

**Table 2:** Application of NPs coated in CCMs in vaccines in recent 5 years.

CCM Source	NPs Coated	Processing Technology	Application/Outcome	Year	Ref.
B16-OVA (B16 melanoma, mouse)	AECM@PC7A (Antigen-enriched B16 cancer cell membrane coated on STING-activating polymer)	Ultrasonic method	Robust CD8^+^ T cell response, strong anti-tumor immunity, neoantigen specificity, memory generation, and metastasis suppression	2025	[36]
B16-F10 (melanoma, mouse)	SCCM@NA (Senescent cancer cell membrane-coated on CpG-loaded Mesoporous Silica Nanoadjuvant)	Sonication & Extrusion	Enhanced DC internalization, improved lymph node targeting, robust CD8^+^ T cell activation, strong antitumor immunity, synergy with αPD-1, suppression of metastasis	2024	[37]
CT26 (colon cancer, mouse)	LDH-based nanovaccine coated with CT26 CCM (LGCMB)	Extrusion method	Activated dendritic cells, enhanced CD8^+^ T cell response, strong CRC tumor suppression	2023	[35]
CT26 (colon cancer, mouse)	PLGA/gambogic acid(GA) NPs coated with CT26 CCM (CCM-PLGA/GA)	Extrusion method	Dual mechanism: direct GA-mediated killing & immune modulation by CCM antigens	2023	[39]
RM-1 (prostate cancer, mouse)	PMBEOx-COOH NPs loaded with R837 and coated with RM-1 CCM (SCNPs/R837)	Extrusion method	Triggered strong lymph node DC activation; synergized with anti-PD1 to suppress prostate tumors	2023	[40]
4T1 (breast cancer, mouse)	Aluminum phosphate NPs loaded with CpG and coated with B16-F10 CCM (APMC)	Extrusion method	Significantly reduced tumor size (avg. ≤ 646 mm) vs. control; prolonged survival with combo therapy	2022	[41]
ID8 (ovarian cancer, mouse)	CaCO_3_ NPs loaded with Dox in the core and Ce6 in the ID8 CCM shell(MC/Dox/Ce6)	Extrusion method	Strongest CD3^+^/CD8^+^ fluorescence, smallest tumors via ROS-PDT and ICD induction	2022	[42]
C1498 (AML, mouse)	PLGA NPs loaded with CpG-ODN 1826 and coated with C1498 CCM (AMC NPs)	Ultrasonic method	Increased survival to 4.4 weeks vs. 2.7 weeks in WCL group; 85% survival at week 21	2022	[33]
4T1 (breast cancer, mouse)	PLGA NPs loaded with R837 and coated with 4T1 CCM (CCMsP@R837)	Extrusion method	75% mice survived >50 days; increased CD8^+^ T and memory T cells; decreased Tregs	2021	[43]
B16-OVA (melanoma, mouse)	B16 Cell membrane vesicles (CMVs) with CpG and dendritic cell (DC)-specific intercellular adhesion molecule (ICAM)-3 grabbing nonintegrin (DC-SIGN)-targeting aptamer	Extrusion method	Robust antitumor response via CpG/TLR9 and DC-SIGN-mediated DC targeting	2021	[44]
4T1 (breast cancer, mouse)	Calcium oxide NPs loaded with DOX and Ce6, coated with 4T1 CCM	Extrusion method	Minimal drug release at pH 7.4; dual tumor inhibition (primary: ≤126 mm; distant: ≤89 mm)	2021	[45]
B16-F10 (melanoma, mouse)	Aluminum phosphate NPs loaded with CpG, coated with B16-F10 CCM	Extrusion method	Extended median survival to 29 days; strongest tumor suppression among groups	2020	[34]

## 3. Fabrication and Characterization of CCM-Coated NPs

**Figure 2:**
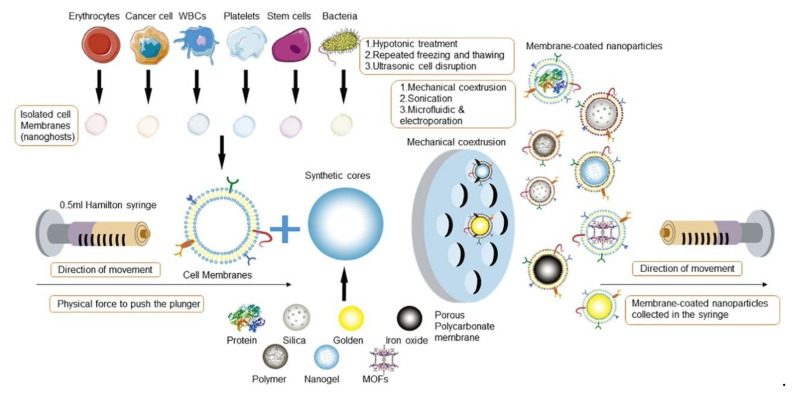
In the physical coextrusion approach for membrane coating fabrication, isolated cell membranes are first obtained using methods such as hypotonic treatment, repeated freeze–thaw cycles, or ultrasonic disruption. The synthetic cores are then coextruded through a porous polycarbonate membrane to achieve membrane coating. Adapted with permission. Ref. [47] Copyright 2019, Nanomicro Lett.

**Figure 3:**
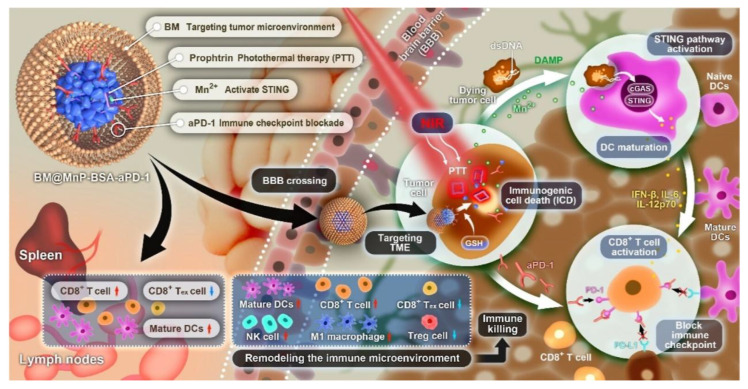
Microglia Membrane–Inspired Nanoplatform with Multiple Synergistic Effects for Tumor Microenvironment Remodeling and Enhanced Glioblastoma Immunotherapy. Ref. [69] Adapted with permission. Copyright 2024, ACS Nano.

The fabrication of CCM-NPs generally follows a three-step process: (1) extraction of cancer cell membranes, (2) fusion of the extracted membranes with NPs (Figure 2), and (3) characterization of the resulting membrane-coated NPs to verify their structural integrity and biological functionality. Further modifications may be applied to enhance targeting efficiency, prolong circulation time, or improve therapeutic efficacy [46].

### 3.1. Cell Membrane Extraction

The extraction of cancer cell membranes involves separating the plasma membrane from intracellular components while maintaining its structural and functional integrity. This is crucial because key membrane proteins and lipids responsible for homotypic targeting and immune evasion must remain intact for the biomimetic NPs to function effectively. To obtain a sufficient quantity of cell membranes, large numbers of cancer cells are harvested from culture or biological samples, followed by cell lysis to remove intracellular contents [48,49,50]. A variety of approaches have been employed to achieve this objective, including freeze–thaw cycles [49,51,52], electroporation [53], and osmosis-based lysis combined with mechanical homogenization [49,54].

The freeze–thaw method involves freezing cells at −80 °C followed by thawing at room temperature or 37 °C through multiple cycles. During the process of forming and rupturing ice crystals, damage is caused to the cell membranes, allowing the expulsion of cytoplasmic contents while retaining the membranes. This method is particularly suitable for non-nucleated cells (e.g., red blood cells and platelets) but may compromise membrane integrity, protein stability, and overall functionality when applied to nucleated cells [49,52,55].

By exposing the cell membranes to strong electric fields, electroporation disrupts the integrity of the cell membrane, leading to temporary loss of semi-permeability, pore formation, and the release of intracellular components [53]. However, excessive electrical stress can cause irreversible membrane damage, protein denaturation, and disruption of lipid asymmetry. To minimize these adverse effects, electroporation conditions must be carefully optimized, as overly harsh parameters may compromise the natural function and stability of the membrane [53,56].

An osmosis-based, hypotonic solution (also known as a homogenizer solution) is commonly used to lyse cancer cells by inducing osmotic swelling, followed by mechanical disruption with a homogenizer [49]. There are several methods of segregating intracellular macromolecules, membranes, and nuclei of the cells using discontinuous gradient centrifugation [48,57], and most efficiently by using continuous gradient centrifugation, which allows the recovery of a membrane-rich fraction that is washed with isotonic buffer to yield membrane vesicles. Depending on the desired size of these vesicles, the membranes can be further processed using sonication or extrusion through polycarbonate membranes for better size control [49]. A notable difference between cancer cell lysates and non-nucleated cells is that cancer cells should usually undergo milder lysis conditions and higher ultracentrifugation speeds. Due to variations in phospholipid bilayer fluidity and cell size, different eukaryotic cell types may require specific extraction methods to optimize membrane yield and functionality [55].

### 3.2. Membrane-NP Fusion and Coating

Several approaches can be employed to coat NPs with cell membranes, but the most common ones are physical extrusion, sonication, and microfluidic coating.

Physical extrusion is a widely used technique for coating NPs with CCMs. In this method, CCMs and NP cores are mixed at a specific ratio and forced through a polycarbonate membrane using an extruder. The mechanical force applied during extrusion disrupts the CCM structure, enabling the membranes to reassemble around the NPs, forming a stable core–shell structure [58]. For example, Feng et al. [59] successfully coated boron nitride nanospheres (HM-BNs) with human cervical cancer cell membranes. The cell membrane suspension was mixed with boron nitride NPs in a 1:1 volume ratio, followed by 30 cycles of extrusion in a micro-extruder. The resulting HM-BNs were then collected via centrifugation at 13,500× *g* rpm for 12 min and lyophilized at room temperature to maintain stability. CCMs-NPs produced by the extrusion method are known for their robustness and reproducibility, consistently exhibiting uniform size, stable zeta potential, and controlled membrane thickness. Sonication is another commonly used method, where ultrasonic energy disrupts membrane vesicles, allowing spontaneous self-assembly into core–shell nanostructures [57,60]. Compared to physical extrusion, this technique usually minimizes the loss of material and provides a simpler coating approach.

Despite these advantages, both physical extrusion and sonication have limitations, including labor-intensive procedures, time inefficiencies, and batch-to-batch variability. To address these issues, microfluidics has emerged as an advanced and highly controlled method for membrane coating, enabling the rapid and precise mixing of NPs with membrane vesicles. Unlike traditional techniques, microfluidics can integrate electroporation and controlled fluid dynamics to enhance membrane fusion efficiency. Several studies have successfully demonstrated microfluidic-based coating techniques, including the functionalization of magnetic NPs with red blood cell membranes [53]. Liu et al. [61] further refined this approach by developing a microfluidic-sonication hybrid system, combining acoustic pulses and hydrodynamic mixing to simultaneously coat NPs with exosome membranes, CCMs, and lipid layers. The resulting biomimetic NPs exhibited superior biocompatibility and therapeutic performance. However, optimizing parameters such as pulse voltage, duration, and flow velocity remains critical for achieving consistent results.

Electrostatic self-assembly relies on charge interactions between the negatively charged membrane and the positively charged NP core, facilitating spontaneous membrane fusion. By leveraging the electrostatic interactions, a stable core–shell structure can be achieved, where the membrane components are correctly oriented, preserving their biological functionality [49,52,62]. This method is simple and effective, but a precise charge balance is essential to prevent NP aggregation and ensure a uniform, stable coating.

To maximize efficacy, each coating method must be optimized based on factors such as NP composition (liposomal, polymeric, or metallic), therapeutic applications, and scalability for clinical translation.

### 3.3. Membrane-Coated NP Characterization

Characterization of CCM-NPs is essential to confirm successful membrane coating and to ensure that their physicochemical and biological properties meet therapeutic requirements [47]. After membrane functionalization, nanoparticles often undergo changes in diameter, surface charge, morphology, and protein composition, all of which need to be carefully evaluated. Transmission electron microscopy (TEM) and cryo-electron microscopy (cryo-EM) are widely used to visualize the membrane coating, confirm the presence of a core–shell structure, and assess uniformity at the nanoscale [49,63]. Complementary to imaging, dynamic light scattering (DLS) provides data on hydrodynamic size and polydispersity index (PDI), which are critical for formulation consistency, while zeta potential measurements reveal surface charge variations that indicate successful wrapping and predict colloidal stability under physiological conditions [64].

In addition to physicochemical characterization, biochemical validation is required to confirm the retention of functional membrane components. Sodium dodecyl sulfate-polyacrylamide gel electrophoresis (SDS-PAGE) and Western blotting are routinely used to detect membrane-specific proteins, while proteomic profiling and lipidomic analysis allow for comprehensive identification of retained proteins, lipids, and adhesion molecules [26,46]. Advanced approaches such as immunogold labeling with TEM, flow cytometry, and enzyme-linked immunosorbent assays (ELISA) can further verify the orientation and bioactivity of membrane proteins, which is critical since inverted or denatured proteins may compromise homotypic targeting and immune evasion functions [65]. Fluorescence resonance energy transfer (FRET) and Förster-based assays are also employed to assess fusion efficiency between the membrane and nanoparticle cores, providing additional evidence for coating integrity [66].

Despite these advances, the characterization of CCM-NPs still faces several important challenges. One major limitation is the lack of standardized protocols, which makes it difficult to compare results across different laboratories. Moreover, batch-to-batch variability in membrane isolation and coating efficiency introduces inconsistency, and correlating in vitro characterization results with in vivo performance is difficult due to the complexity of the tumor microenvironment [67]. Addressing these challenges will be important for the reliable evaluation and clinical translation of CCM-NPs [23].

## 4. Applications of CCM-NPs in Various Cancer Therapies

### 4.1. Glioblastomas (GBM)

GBM is an aggressive and highly heterogeneous brain tumor with limited treatment options. The blood–brain barrier (BBB) restricts drug penetration, while the TME suppresses immune responses, making it difficult for conventional therapeutics to achieve significant efficacy. CCM-NPs offer an effective strategy by utilizing homologous tumor membranes, which enable them to cross the BBB, recognize tumor cells, and enhance drug delivery [68]. Table 3 highlights various CCM sources, NP formulations, processing technologies, and therapeutic applications in GBM research. Notably, the use of U87MG and U251 glioma cells as CCM sources has been widely adopted due to their ability to enhance NP retention within GBM tumors. Fan et al. [69] developed a microglia membrane biomimetic nanoplatform (BM@MnP-BSA-aPD-1) designed to penetrate the BBB and achieve targeted therapy for GBM. This nanocarrier leverages the biological properties of microglia membranes to enhance BBB permeability while enabling precise drug delivery within the TME (Figure 3). The study demonstrated that this nanosystem effectively activates the cGAS-STING pathway, induces ICD, and synergizes with photothermal immunotherapy (PTT) to modulate the immunosuppressive TME and strengthen antitumor immune responses. This strategy offers a novel, chemotherapy-free approach to GBM immunotherapy and holds significant potential for clinical translation. Ren et al. [70] developed a multifunctional biomimetic NP platform that encapsulates graphene quantum dots (GQDs) and DOX within homologous CCMs (BV2 cells) for targeted GBM therapy. The GQDs act as photothermal agents, possessing stable fluorescence and excellent photothermal conversion properties. In addition to preventing DOX leakage, the adhesion factors on the CCMs enhance the uptake of NPs by tumor cells, while the high temperatures generated by near-infrared laser irradiation facilitate the release of DOX, minimizing adverse effects on normal tissues. The study demonstrated that GQDs/DOX@CCMs actively targeted BV2 mouse microglial cells in vitro, resulting in higher cellular uptake and improved cancer cell killing efficiency via combined chemophotothermal treatment. This biomimetic NP platform offers a strategic reference for precision treatment in tumors.

### 4.2. Breast Cancer

Breast cancer remains one of the most prevalent malignancies worldwide, with chemotherapy as the primary treatment [77]. However, poor drug accumulation at tumor sites and severe systemic toxicity limit the effectiveness of current therapies [78]. Biomimetic NPs coated with breast cancer cell membranes have emerged as a targeted delivery platform to improve drug bioavailability and reduce off-target effects (Table 4). Xiong [79] et al. developed a biomimetic tumor cell membrane-coated NP delivery system for combination therapy of triple-negative breast cancer (TNBC). This system integrates NIR-II photothermal therapy, chemotherapy, and immunotherapy, leveraging TNBC cell membrane camouflage for efficient targeted drug delivery. The study demonstrated that AgPP@P@M NPs could induce ICD, trigger the release of damage-associated molecular patterns (DAMPs), and synergize with PD-L1 inhibitors to enhance the antitumor immune response. Furthermore, in a TNBC lung metastasis model, the nanoplatform effectively reduced lung metastases by 51.2% and significantly prolonged tumor remission. This innovative nanodelivery strategy offers a promising approach for efficient TNBC immunotherapy and holds great potential for clinical translation. Wang [80] et al. developed a breast cancer cell membrane-coated, siRNA-functionalized Au/MnO_2_ radiosensitizing nanoplatform (R&F@Au/MnO_2_-CM) to synergistically enhance radio-immunotherapy for breast cancer. This nanoplatform leverages 4T1 breast cancer cell membranes for homotypic targeting, while Au/MnO_2_ components improve X-ray absorption and alleviate tumor hypoxia. Simultaneously, PD-L1 siRNA efficiently downregulates PD-L1 expression in tumor cells, modulating the immunosuppressive microenvironment. In a 4T1 breast cancer mouse model, the system enabled precise tumor identification via NIR-II fluorescence imaging and enhanced radiosensitivity upon X-ray exposure. Furthermore, it promoted CD8^+^ T-cell infiltration, significantly inhibited tumor growth, and prolonged survival in treated mice. This study presents a novel nanomedicine strategy for breast cancer radio-immunotherapy, offering promising potential for clinical translation.

Han et al. [81] developed a biomimetic nanosphere drug delivery system coated with CCMs. This system consists of two components: paclitaxel-loaded poly (lactic acid) NPs (PPNs) and a biomimetic shell derived from 4T1 mouse breast cancer cell membranes, referred to as CPPNs. The study demonstrated that CPPNs exhibited significant in vitro antitumor efficacy against 4T1 cells and effectively accumulated within tumor tissues due to the homotypic targeting capabilities conferred by the CCMs. Moreover, CPPNs effectively inhibited the growth of in situ 4T1 breast cancer cells in mouse models while displaying good biocompatibility.

In addition to coating NPs with CCMs, Gong et al. [82] enhanced targeting ability by encapsulating NPs with hybrid membranes derived from immune cells and cancer cells. They extracted membranes from RAW264.7 macrophages and 4T1 breast cancer cells using extrusion and centrifugation methods. The hybrid membrane, designated RAW-4T1, was created by combining the membranes from both cell types through ultrasound treatment. Subsequently, the core doxorubicin-loaded poly (lactic-co-glycolic acid) (DPLGA) was encapsulated with the RAW-4T1 membrane, resulting in DPLGA@[RAW-4T1] NPs. This formulation demonstrated low systemic toxicity and no significant myocardial damage in a 4T1-luc tumor model, leading to an extended survival period. The 4T1 membrane coating enabled DPLGA@[RAW-4T1] NPs to achieve targeted delivery to cancerous sites.

**Table 4:** Studies on NPs Coated by breast cancer cell membrane in the past 5 years.

CCMs Source	NPs Coated	Processing Technology	Application/Outcome	Year	Ref.
4T1 (breast cancer, Mouse)	CAMD@CM(coating COF nanospheres with 4T1 cell membrane and loading with Dox)	Ultrasonic method	ICD induction, antigen delivery, DC activation, suppressed tumor growth and metastasis	2025	[83]
4T1 (breast cancer, Mouse)	MSF@CCM (mesoporous silica-loaded FeOOH core coated with 4T1 cell membrane)	Extrusion method	Enhanced tumor accumulation, immune evasion, and ultrasound-mediated deep tumor penetration; triggered ferroptosis and achieved 96.5% tumor growth inhibition.	2025	[84]
4T1 (breast cancer, Mouse)	DTX@CHMSN (docetaxel-loaded HMSN coated with 4T1 cell membrane)	Extrusion method	Improved water solubility of docetaxel, homologous targeting and immune evasion, enhanced accumulation at tumor site, reduced systemic toxicity, and improved therapeutic efficacy.	2024	[85]
4T1(breast cancer, Mouse)	R&F@Au/MnO_2_-CM (siRNA & Au/MnO_2_ nanosensitizer with 4T1 membrane)	Extrusion method	Enhanced radiotherapy and immune activation, prolonging survival.	2024	[80]
4T1(breast cancer, Mouse)	TNBC membrane-coated NIR-II/chemo/PD-L1 inhibitor nanoplatform	Extrusion method	Suppressed lung metastasis by 51.2% and extended tumor remission.	2024	[79]
4T1(breast cancer, Mouse)	Fe_3_O_4_ NPs with ICG and R837, coated with hybrid TRM membrane	Ultrasonic method	Amplified photothermal/Fenton effect and activated CD8^+^ T cell immunity.	2023	[86]
4T1(breast cancer, Mouse)	IR-1048 liposomes coated with 4T1 membrane	Extrusion method	Achieved 96.16% tumor cell killing and significantly inhibited tumor growth.	2022	[87]
4T1(breast cancer, Mouse)	PB & DTX/R837-loaded PLGA nanospheres with 4T1 membrane	Ultrasonic method	Boosted tumor cell uptake and increased CTL infiltration from 17.3% to 35.5%.	2021	[28]
4T1(breast cancer, Mouse)	Hybrid membrane-coated Dox-loaded poly(lactic-co-glycolic acid) (PLGA) NPs (DPLGA@[RAW-4T1] NPs)	Ultrasonic method	Achieved 88.9% anti-metastasis efficacy in a lung metastasis model.	2020	[82]
4T1(breast cancer, Mouse)	CPPNs (PTX-loaded PLA NPs with 4T1 membrane)	Extrusion method	Enhanced anti-tumor efficacy and reduced PTX toxicity in vitro and in vivo.	2020	[81]

### 4.3. Other Tumors

In addition to brain cancer and breast cancer, CCM-coated NPs have been applied to the treatment of various tumor types (Table 5), primarily through the encapsulation of drugs using cancer cell membranes or hybrid membranes formed by cancer cells and other cell types, enabling effective drug delivery to tumor sites and inhibiting tumor growth. For example, Wang et al. [88] developed a hybrid membrane composed of outer membrane vesicles (OMVs) and B16-F10 cancer cell membranes, which encapsulated hollow polydopamine NPs (HPDA) on their surface, resulting in HPDA@[OMV-CC] NPs. The photothermal therapy mediated by HPDA induces cancer cell apoptosis and necrosis, leading to the release of tumor-associated antigens and the induction of an antitumor immune response. Studies showed that mice injected intravenously with HPDA@[OMV-CC] NPs demonstrated uniform targeting of melanoma. The combination of OMV-based immunotherapy with HPDA-mediated photothermal therapy effectively treated melanoma without significant adverse effects.

## 5. Summary and Outlook

CCM-NPs represent a rapidly advancing area in biomimetic nanomedicine. By integrating tumor-derived membranes with diverse nanoparticle cores, these systems combine homologous targeting, immune evasion, and enhanced penetration of biological barriers. As summarized in this review, CCM-NPs show promise not only in precision drug delivery but also as versatile platforms for immunotherapy, photothermal/photodynamic therapy, and cancer vaccination. Their ability to retain tumor antigens and to interact with the tumor microenvironment highlights their potential as next-generation agents in precision oncology. Nevertheless, simply cloaking nanoparticles with unmodified membranes is unlikely to fully address tumor heterogeneity and immunosuppressive barriers. Future progress will depend on strategic engineering of CCMs to endow nanoparticles with multifunctional properties that enhance their therapeutic performance and overcome biological limitations.

Recent advances in synthetic biology and genetic engineering offer new opportunities to tailor CCMs beyond their native state. For example, genetically editing donor cancer cells to overexpress costimulatory molecules, cytokines, or tumor neoantigens could generate membranes that both guide nanoparticles to tumors and actively modulate immune responses. Similarly, CRISPR-based knockdown of immune-evasive ligands (e.g., PD-L1, FasL, or CD47) could minimize undesired immunosuppression while preserving targeting capacity. Beyond genetic modification, chemical and biomaterial engineering approaches, such as conjugating membranes with adjuvants, checkpoint inhibitors, or extracellular matrix-penetrating peptides, can further enhance therapeutic specificity and efficacy [98].

In addition, the hybrid membrane strategies that integrate cancer cell membranes with those from immune or stromal cells could integrate complementary functions. For instance, dendritic cell/tumor membrane hybrids may simultaneously present tumor antigens and provide costimulatory signals, functioning as potent nanovaccines. Likewise, combining cancer cell membranes with endothelial or stromal membranes could improve navigation through biological barriers, expanding applications in solid tumors with dense extracellular matrices.

However, the application of CCM-NPs remains at the preclinical stage, and no clinical trials have been reported yet. Several key challenges impede their translation to the clinic. First, safety concerns remain paramount. During the extraction of CCMs, residual nuclear components, DNA, or oncogenic proteins from donor cancer cells may contribute to tumor progression in patients, raising concerns about the clinical safety of CCM-coated NPs. This risk underscores the need for rigorous purification strategies (e.g., enzymatic digestion, gradient centrifugation, and nuclease treatment) combined with standardized quality control assays to ensure the complete removal of genetic material before clinical use. Second, manufacturing scalability and reproducibility are major hurdles. Current coating methods, such as extrusion and sonication, require significant time and labor, and often compromise batch-to-batch consistency and membrane orientation. Innovative manufacturing platforms, such as automated microfluidic chips and continuous-flow coating systems, are being developed to enhance throughput, reproducibility, and homogeneity of CCM-NPs [99]. Third, immunogenicity and donor variability pose additional barriers. Allogeneic CCMs may elicit host immune responses, while autologous CCMs are limited by patient-specific variability and availability. Using standardized “universal” tumor cell lines is one potential strategy to mitigate these issues, which provide a consistent membrane source with minimal patient-to-patient variability and lower immunogenic risk. Generating hybrid membranes that combine tumor-derived components with non-immunogenic materials and silencing immunogenic antigens are another two strategies to reduce immune recognition and prolong nanoparticle circulation. Fourth, stability and storage conditions also limit clinical feasibility, as CCMs are prone to degradation. Developing optimized formulations with stabilizing excipients, lyophilization methods, and validated cold-chain logistics is critical. Finally, regulatory approval requires robust guidelines not only for membrane sourcing, characterization, and potency assays but also for comprehensive long-term biosafety evaluations, including biodistribution, immunotoxicity, and genotoxicity in GLP-compliant models. Addressing these barriers will require a multifaceted strategy that integrates standardized purification and quality control pipelines, scalable GMP-compatible manufacturing, rational engineering of donor membranes, improved formulation and storage stability, and rigorous preclinical safety evaluation. Such combined approaches are expected to accelerate the safe and effective clinical translation of CCM-NPs.

In conclusion, CCM-NPs demonstrate the power of biomimetic design to advance cancer treatment. Their successful translation from bench to bedside will depend on interdisciplinary efforts to overcome hurdles in manufacturing, safety, and patient-specific design. Ultimately, these nanoparticles represent a promising pathway toward more effective and personalized cancer therapies [100,101,102].

## Figures and Tables

**Table 1:** Summary of representative cell membrane coatings for nanoparticle-based cancer therapy.

Membrane Source	Main Advantages	Main Limitations
Cancer cell membrane (CCM)	Enables homotypic targeting through tumor antigen recognition; carries immune evasion proteins (e.g., CD47); improves tumor accumulation and selective uptake	Applicable mainly to cancer settings; potential safety concerns from oncogenic proteins; variability in membrane composition and scalability issues
Red blood cell (RBC) membrane	Readily available; well-established isolation methods; strong immune evasion and prolonged circulation time	Does not provide tumor-specific targeting; limited ability to direct nanoparticles to tumor tissues
Platelet membrane	Natural adhesion to damaged vasculature and circulating tumor cells; contributes to immune evasion; useful in metastasis prevention	Limited availability; possible pro-thrombotic activity; less tumor selectivity compared with CCM
Immune cell membrane (e.g., macrophage, T cell, NK cell)	Intrinsic affinity for inflammatory and tumor microenvironments; potential to modulate immune response; can facilitate tissue penetration	Limited cell sources; risk of immunogenicity; functional properties depend on immune cell type
Stem cell membrane	Tropism toward tumor and injured tissues; possesses immune evasion properties; potential for regenerative applications	Safety concerns related to stemness-associated factors; technical challenges in large-scale preparation

**Table 3:** Studies of NPs Coated by GBM cell membrane in recent 5 years.

CCMs Source	NPs Coated	Processing Technology	Application/Outcome	Year	Ref.
U87-MG (GBM cell, human)	Carboxylate-modified micro-sphere FluoSpheres^®^ (PS-NPs) were coated with the isolated U87-MG Cell membrane	Extrusion method	Enhance tumor targeting and BBB penetration for improved glioblastoma therapy.	2025	[71]
U87-MG (GBM cell, human)	Amphiphilic CB [7]-PEG-Ce6 micelles loaded with MTIC	Extrusion method	Crosses BBB, targets TME, enhances metal immunotherapy & PTT, blocks immune checkpoints	2024	[69]
U87-MG (GBMU87 MG cell, human)	HM-NPs@G: Gboxin-loaded NPs coated with hybrid cancer cell-mitochondria membrane	Extrusion method	Prolongs Gboxin circulation, improves BBB permeability and tumor accumulation	2023	[72]
U251 (Glioma cells, human)	M@HLPC NPs: Self-assembled Hb, LOX, CPO-Ce6 particles coated with U251 membrane	Extrusion method	Strong anti-tumor efficacy via LA metabolic therapy & chemiexcited PDT in CDX & PDX models	2022	[73]
BV2 (microglia cell, mouse)	GQDs/DOX@CCMs: Graphene QDs & DOX coated with BV2 CCM	Extrusion method	Stable for 7 days at ~34 °C; 2x fluorescence in BV2 vs. MCF-7; effective for chemophotothermal therapy	2022	[70]
C6 (microglia cell, mouse)	DNS-[C6&DC]m: DTX nanosuspension coated with hybrid C6 & dendritic cell membrane	Ultrasonic method	Significantly prolonged survival (65 d) vs. DTX (37 d) and DNS (42 d)	2021	[74]
C6 (microglia cell, mouse)	HCPT-NS/CCM:10-hydroxycamptothecin nanosuspension camouflaged with C6 CCM	Ultrasonic method	2x DiR fluorescence in tumors; significantly extended survival vs. saline/HCPT/HCPT-NS	2021	[75]
C6 (Glioblastoma cells, rat)	PEI25k/pDNA complexes coated with C6 CCM	Ultrasonic method	Reduced toxicity, higher HSVtk gene expression	2021	[76]

**Table 5:** Applications of other CCM-coated NPs in the past 5 years.

CCM Source	NPs Coated	Coating Method	Application/Outcome	Year	Ref
SGC-7901 (gastric cancer, human) or MFC (gastric cancer, mouse)	PLGA-STM-TAT and PLGA-STM TAT@CCM-YSA	Extrusion method	Tumor targeting, m6A modulation, enhanced anti-gastric cancer immunity	2025	[89]
CNE-2 (Nasopharyngeal carcinoma (NPC), human)	PAMAM dendrimer loaded with DOX, coated with CNE-2 CCM	Ultrasonic method	Prolonged circulation, tumor targeting, systemic anti-NPC efficacy	2024	[90]
AGS (gastric cancer, human)	IRCB@M: hybrid NP inhibiting glutamine metabolism and enhancing ROS-mediated PDT	Extrusion method	Dual-pathway PDT booster via glutamine metabolism inhibition	2024	[91]
HeLa (cervical cancer, human)	HMnO2 NPs loaded with ICG, coated with HeLa CCM	Extrusion method	Strong NIR-triggered antitumor effect with high biocompatibility	2024	[92]
U14 (cervical cancer, mouse)	(dexamethasone) Dex@PLGA-CM	Ultrasonic &Extrusion method	TME modulation, homologous tumor targeting, enhanced Doxil penetration, improved anti-gynecologic cancer efficacy	2023	[93]
K7M2 (osteosarcoma, mouse)	MnO_2_ NPs, functionalized with K7M2 CCM	Extrusion method	Reduced tumor volume; prolonged survival > 55 days	2022	[94]
ID8 & RBC (hybrid, ovarian, mouse)	Fe_3_O_4_ magnetic NPs loaded with ICG, coated with hybrid ID8 ovarian and RBC membranes	Ultrasonic method	ICG accumulation 2.9x higher in hybrid IRM vs. single-membrane NPs	2021	[95]
TE10 (esophageal cancer, human)	PLGA NPs co-loaded with DOX and curcumin, coated with PEGylated TE10 CCM	Extrusion method	Prolonged tumor suppression, reduced cardiotoxicity	2021	[96]
KB (oral cancer, human)	Gold nanorods coated with KB oral cancer cell membranes (GNR@Mem)	Extrusion method	4.7x higher uptake; 95.6% inhibition with PTT & RT	2020	[97]
B16-F10 & OMVs (melanoma, mouse)	Hollow polydopamine NPs coated with hybrid OMVs and B16-F10 melanoma membranes	Ultrasonic method	87x targeting specificity; 45-day survival post NIR-PTT	2020	[88]

## Data Availability

Not applicable.
